# Correlation between serum ANCA and cancer: a decade-long retrospective analysis of an Italian cohort

**DOI:** 10.3389/fimmu.2025.1631498

**Published:** 2025-08-27

**Authors:** Enrico Brunetta, Lorenzo Del Moro, Marco Folci, Giacomo Ramponi, Davide Sacchet, Renato Alberto Sinico, Carlo Selmi

**Affiliations:** ^1^ Rheumatology and Clinical Immunology, IRCCS Humanitas Research Hospital, Milan, Italy; ^2^ Department of Biomedical Sciences, Humanitas University, Milan, Italy; ^3^ Fondazione Poliambulanza Istituto Ospedaliero, Brescia, Italy; ^4^ Dipartimento di Scienze Cliniche e di Comunità, Dipartimento di Eccellenza 2023–2027, Università degli Studi di Milano, Milan, Italy; ^5^ IRCCS Humanitas Research Hospital, Milan, Italy; ^6^ Nephrology and Dialysis Unit, IRCCS Humanitas Research Hospital, Milan, Italy

**Keywords:** ANCA, ANCA-associated vasculitides, cancer, ANCA-negative, vasculitis, autoantibodies, cancer risk, autoimmune biomarkers

## Abstract

**Background:**

Serum antineutrophil cytoplasmic antibodies (ANCA) are autoantibodies primarily linked with ANCA-associated vasculitides (AAV) but associated diseases include also other autoimmune diseases, infections, and malignancies. However, the association of ANCA with cancer risk remains undefined, especially in patients without AAV.

**Objective:**

The aim of this study was to identify the incidence of malignancies in a large cohort during a 10-year period and to compare the incidence in ANCA-positive versus ANCA-negative subjects.

**Methods:**

A retrospective cohort study involving 6285 subjects who underwent ANCA testing was performed at Humanitas Research Hospital (ICH) (Rozzano, Italy) from 2007 to 2017, regardless of the clinical indication for ANCA testing. After retrieving comorbidity data from electronic health records, we classified chronic conditions into six major categories and generated a propensity score including age, gender, time of blood draw, and comorbidities. A 1:2 matching was performed, yielding a final cohort of 214 ANCA-positive and 319 ANCA-negative subjects. The ICH Cancer Diagnosis Registry was used to assess cancer incidence. Competing risk regression was performed using the Fine & Gray model to estimate cancer risk associated with pANCA positivity. The cANCA-positive group (n = 44) was excluded from regression analysis due to the absence of cancer events.

**Results:**

During a mean follow-up of 3.9 years, 43 patients had a diagnosis of cancer (9/214 ANCA-positive patients, and 34/319 ANCA-negative patients; Chi-square p 0.007). In the pANCA-positive subgroup, no significant difference in cancer incidence was observed compared to ANCA-negative subjects. ANCA-negative subjects did not have significantly longer survival after cancer diagnosis compared with pANCA-positive patients (median survival: 203 days [IQR 120-309] vs. 266 days [IQR 124-580]). In competing risk regression analysis, no association was found between positive pANCA and cancer risk (HR 0.50; CI 95% 0.240–1.043; p 0.065).

The results were consistent even when AAV cases were excluded (HR 0.53; 95% CI 0.24–1.16).

**Conclusion:**

Isolated ANCA positivity is not associated with a higher incidence of malignancy. There was no significant difference in post-cancer survival between ANCA-negative and pANCA-positive subjects. The absence of malignancies in the cANCA subgroup should be interpreted with caution given the small sample size. These observations should be confirmed by prospective studies, which also should ascertain the possible underlying mechanisms.

## Introduction

Antineutrophil cytoplasmic antibodies (ANCA) are autoantibodies primarily involved in the pathogenesis of a subset of small-vessel vasculitides known as ANCA-associated vasculitides (AAV). AAV includes granulomatosis with polyangiitis (GPA), microscopic polyangiitis (MPA), and eosinophilic granulomatosis with polyangiitis (EGPA) (1, 2). ANCA are detected by indirect immunofluorescence (IIF) and chemiluminescence immunoassay (CLIA), which produce two main patterns: cytoplasmic (cANCA) and perinuclear (pANCA) (3). Proteinase 3 (PR3) and myeloperoxidase (MPO) are the primary targets of the c- and p-ANCA in AAV patients, respectively; PR3 is most commonly associated with GPA, while MPO is more frequently linked to MPA (4). Serum ANCA is also present in a number of other conditions (5), including but not limited to rheumatoid arthritis, systemic lupus erythematosus, ulcerative colitis, autoimmune hepatitis, sclerosing cholangitis, infections, malignancies, and other disorders.

AAVs have been associated with solid cancers, particularly kidney and colon cancer, and hematologic malignancies (6). Several case reports, case series, and a retrospective multicenter study have described an AAV likely triggered by an underlying hematopoietic malignancy (7–12).

Some studies suggest that vasculitis occurring in close temporal association with cancer may constitute from 0.4 to 4.2% of all vasculitis cases (11). The early recognition of hematologic malignancies in AAV is important since lymphoma has been reported to present in a way that mimics vasculitis (13, 14).

Most studies investigating the relationship between AAV and leukemia have reported an increased risk of developing some sort of blood neoplasm in patients who underwent immunosuppressive treatment, especially with cyclophosphamide. At present, no significant role can be attributed to AAV vessel inflammation in stimulating oncogenesis. In a comprehensive review, some studies have focused on other mechanisms that might increase cancer risk in AAV patients and found contradictory evidence regarding a possible shared pathogenesis, concluding that suspicion of a neoplastic process should not be increased in AAV patients (15). Another study found similar results (16). In such a complex scenario, the theoretical ambivalent role of PR3 in GPA and acute myeloid leukemia is noteworthy.

In a previous study by our group (17), we investigated the prognostic significance of ANCA positivity in a large retrospective cohort of Italian patients tested for ANCA for various clinical reasons. According to the research, having pANCA and, to a lesser degree, cANCA was linked to a higher risk of dying from any cause, especially in people who had autoimmune conditions that weren’t considered AAV. The study did not, however, examine cancer incidence as an endpoint or address particular causes of death, such as cancers. Building on these results, the current study focuses on the relationship between ANCA positive and the chance of developing cancer later on, regardless of whether vasculitis has been clinically diagnosed.

Previous studies (6) have evaluated the prevalence of tumors in patients with AAV versus the general population but the same information in patients with the only presence of ANCA (without associated systemic vasculitis) in a large cohort is not clear (18). The role of these antibodies in tumor pathogenesis is unknown, and the absence of a clinical complex context, such as the presence of AAV, can be useful to better understand the possible correlation between them. The objective of this study is to obtain the incidence of malignancy over 10 years in a large Italian cohort of subjects tested for serum ANCA.

## Materials and methods

### Subjects and laboratory tests

Between 2007 and 2017, 6285 patients were tested for serum ANCA at the laboratory of the Humanitas Research Hospital (Rozzano, Italy), the test being ordered by immunologists, rheumatologists, gastroenterologists, neurologists, and other physicians. All sera underwent IIF testing and, if positive, ELISA testing for anti-MPO, anti-PR3, and other ANCA antigens using the ANCA Test System (Immunoconcept, Bordeaux, France), RA-MPO, and RA-PR3 ZENIT (Menarini Diagnostics, Florence, Italy). As described elsewhere (17), specific criteria were used to determine the ANCA result and subjects with more than one ANCA test were only counted once. If all tests were negative, the earliest date of a negative test was recorded while if all tests were positive, the earliest positive test was recorded. If results were mixed, the earliest positive test was recorded. Patients with missing IDs were excluded, as were patients who were not residents of the Lombardia region to ensure complete and consistent data on vital status and cancer diagnoses, available through the Lombardia Regional Health Registry and the ICH Cancer Diagnosis Registry. This approach minimized missing data, enhanced follow-up completeness, and reduced potential detection and surveillance bias, though it may limit generalizability to other regions. The database containing ANCA testing data was merged with the ICH registry of cancer diagnoses. Patients who were diagnosed with cancer prior to ANCA testing were excluded because it was not possible to demonstrate ANCA exposure prior to cancer development.

Patients with low-titer pANCA at IIF, high-titer ANA, and negative MPO/PR3 ELISA were considered false positives. This definition follows the established immunological practice, as a perinuclear ANCA pattern at low titer by IIF, in the presence of a strong ANA signal and negative MPO/PR3 ELISA, is most commonly attributable to ANA interference on ethanol-fixed substrates rather than true ANCA positivity. This interpretation is consistent with the 2017 International Consensus on ANCA Testing (5), which notes that high-titer ANA can interfere with pANCA detection on ethanol-fixed neutrophils. In such cases, when ANA and pANCA titers are comparable, the IIF result should be considered “not determinable” due to the risk of misinterpretation. Therefore, low-titer pANCA in ANA-positive, MPO/PR3-negative samples should be interpreted with caution and may represent false positives.

We retrieved all available diagnostic information from the electronic health records (EHR) of our institution and classified comorbidities into six clinically meaningful categories (cardiovascular, respiratory, metabolic, renal/hepatic, neurologic, and autoimmune/inflammatory diseases) as illustrated in the **Supplementary Table S1**). Using these variables with age, gender, and time of blood draw, we computed a propensity score and performed a 1:2 (ANCA-positive: ANCA-negative) matching to select a demographically and clinically comparable ANCA-negative population. This matching procedure yielded a final matched cohort of 214 ANCA-positive and 319 ANCA-negative subjects.

Data on deaths were obtained from the Italian Health registry system. All patients included in the study had given their informed consent for research use of their data.

### Statistical analysis

This retrospective cohort study analyzed data collected between November 9, 2007 and November 9, 2017. The follow-up period extended from the time of blood draw for ANCA testing to the time of event or November 9, 2017, if no event occurred. The incidence of cancer was the primary outcome studied. The competing risk setting was analyzed to model the incidence of cancer. Non-parametric cumulative incidence curves were calculated, and the Fine & Gray model was fitted to estimate the association between cancer risk and ANCA exposure in terms of sub-hazard ratio (sub-HR). With a power of 0.8, an alpha error of 0.05, and the matched sample size, the study was powered to detect a hazard ratio no smaller than 1.27.

Given the absence of malignancies in the cANCA-positive subgroup (n = 44), this group was excluded from competing-risk regression modeling, as no hazard function could be estimated.

To evaluate the adequacy of the matching procedure, we assessed the balance of baseline covariates between pANCA-positive and ANCA-negative patients before matching. **Supplementary table S2** shows the standardized mean differences and results of t-tests for key variables. Statistical analyses were conducted using Stata 15 and RStudio.

## Results

**Figure 1:**
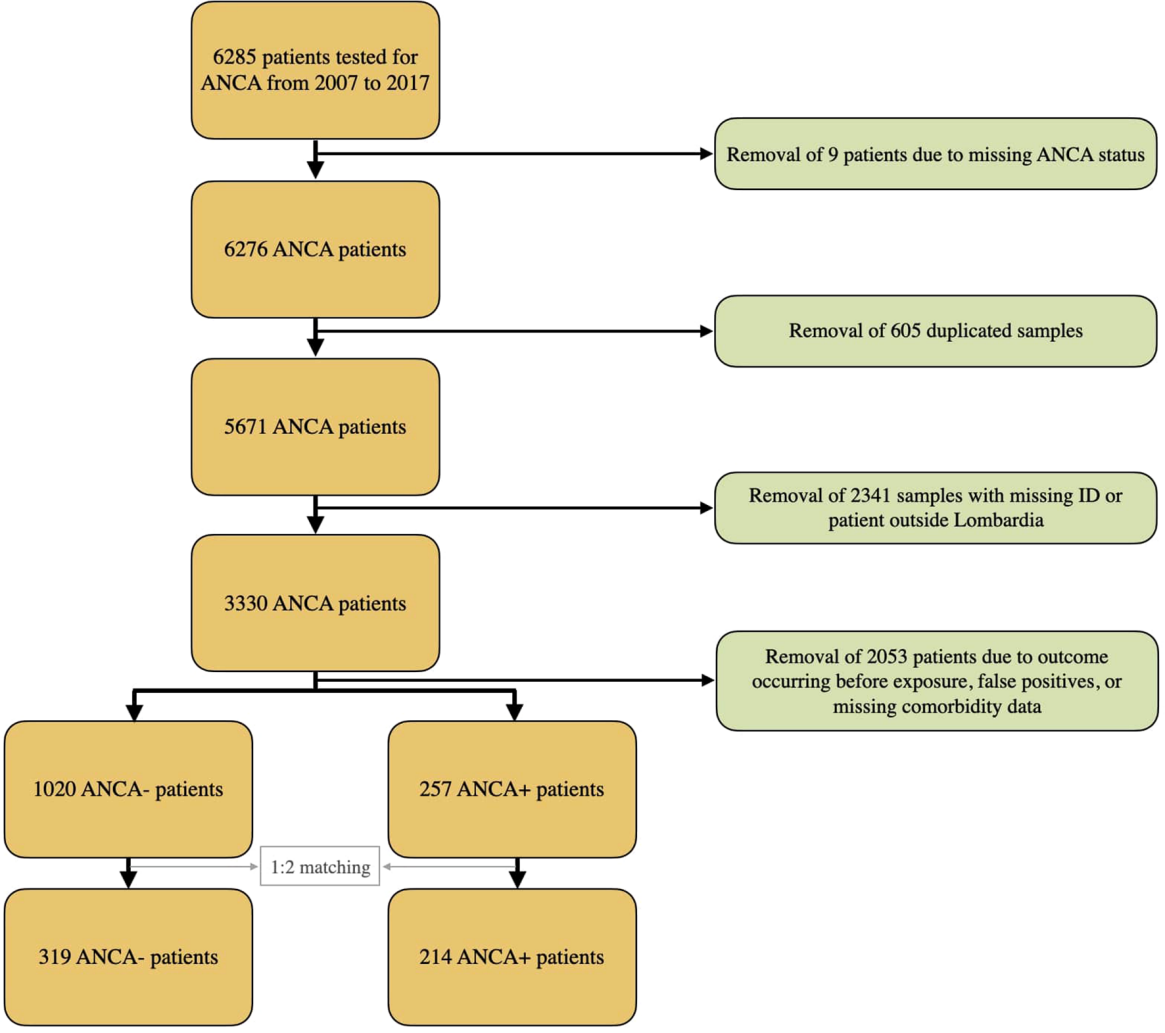
Flowchart of study selection process.

The complete dataset included 5671 subjects (**Figure 1**); among these, 446 subjects tested positive for ANCA, while 5225 tested negative, resulting in an ANCA crude prevalence of 7.86%.

Following the application of exclusion criteria, retained patients were applied a 1:2 propensity score matching based on age, gender, time of blood draw, and comorbidities.

The final matched cohort consisted of 214 ANCA-positive and 319 ANCA-negative subjects. The mean follow-up duration was 3.9 years, corresponding to a total of 2312 person-years at risk.

During the follow-up period, 157 deaths were recorded, while four subjects (three ANCA-negative and one ANCA-positive) were lost to follow-up.

**Table 1** illustrates the number of subjects diagnosed with cancer diagnosed over a 10-year period. During the follow-up, 43 patients developed malignancies (9/214 ANCA-positive patients, and 34/319 ANCA-negative patients; Chi-square p 0.007). Of the 43 subjects with malignancies, 12 died within 10 years, with a mean interval of 351 days between diagnosis and death. Among the 214 ANCA-positive patients in the matched cohort, 44 were cANCA-positive and 170 were pANCA-positive. No cancer cases occurred in the cANCA-positive group during the follow-up period, whereas 9 cases were observed among pANCA-positive patients. Due to the absence of events in the cANCA group, it was not possible to estimate a sub-hazard function, and this group was therefore excluded from competing-risk regression. The mean follow-up duration was similar across the subgroups. Nevertheless, the small sample size of the cANCA subgroup limits the strength of any inference regarding cancer risk in this population.

**Table 1:** Distribution of patients with tumors stratified by ANCA status.

ANCA status	Number of patients with tumors	Total number of patients	Time at risk (years)
ANCA negative	34	319	1358
cANCA-positive	0	44	258
pANCA-positive	9	170	695
total	43	533	2312

This table presents the distribution of patients with tumors stratified by ANCA status. For each group (ANCA-negative, cANCA-positive, and pANCA-positive), the table reports the number of tumor cases, the total number of patients at baseline, and the total person-years at risk. These data provide context for the incidence and time-to-event analyses across different ANCA profiles. Notably, no tumors were observed in the cANCA-positive subgroup.

**Figure 2:**
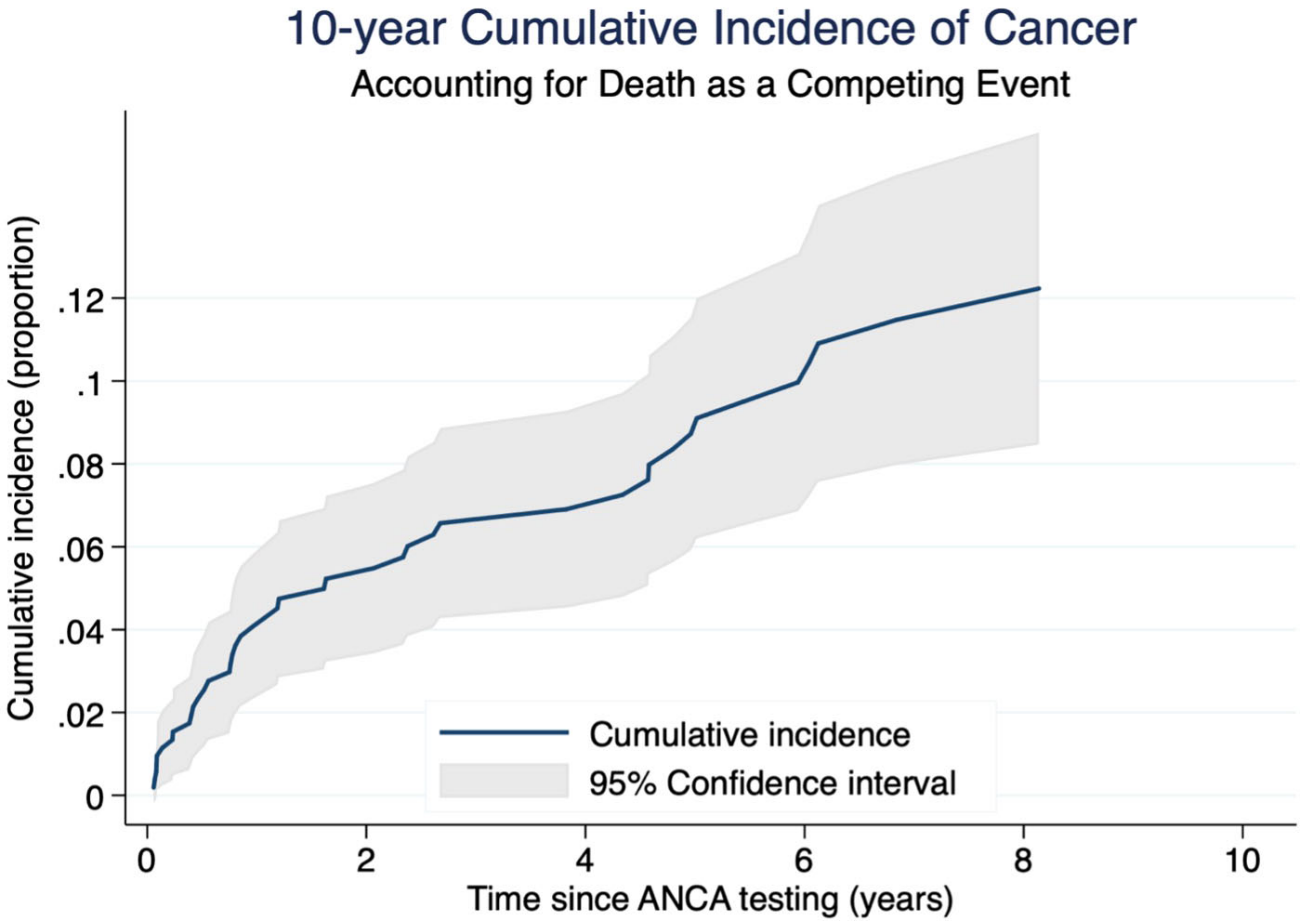
Cumulative incidence of cancer with 95% confidence interval in a competing risks framework. The figure shows the 10-year cumulative incidence of cancer in the cohort, accounting for death as a competing risk. The blue solid line represents the estimated cumulative incidence function over time (cancer as event of interest). The light gray shaded area represents the 95% confidence interval, derived from standard errors of the estimates. Time is measured in years since ANCA testing, and the outcome is expressed as a proportion of cumulative risk.

**Figure 3:**
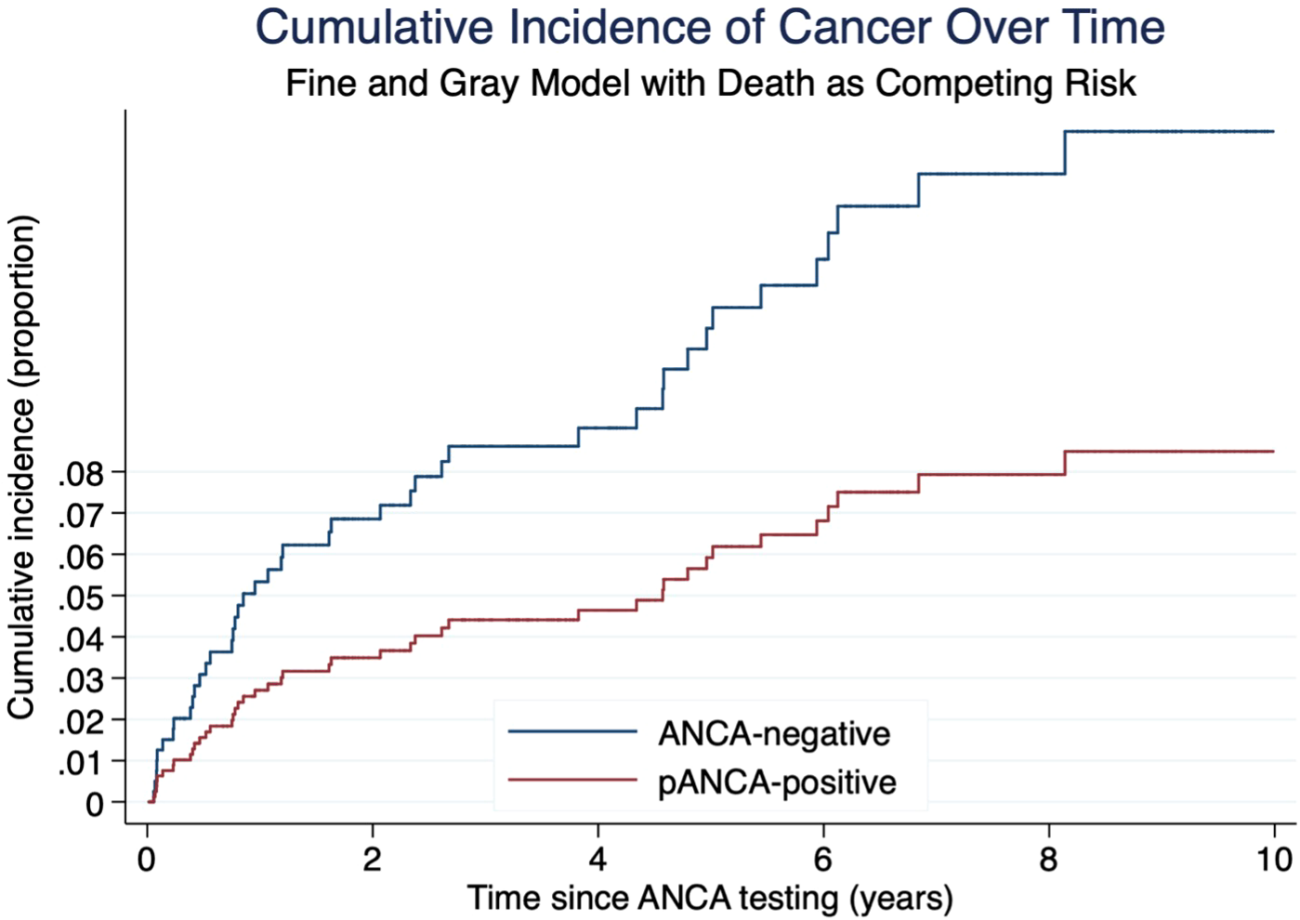
Cumulative incidence of cancer by ANCA status estimated using Fine and Gray competing risk regression. Death was treated as a competing event. Blue line: ANCA-negative group. Red line: pANCA-positive group. Time is expressed in years from ANCA testing. Note: cANCA-positive patients were excluded from this graph due to absence of cancer events.

**Table 2** provides a detailed overview of the diagnosed cancer types. The non-parametric cumulative incidence function (CIF), along with confidence intervals accounting for competing risks (death), is depicted in **Figure 2**. Competing-risks regression, performed using the Fine and Gray method, did not indicate a significantly altered tumor risk in the presence of pANCA (pANCA: HR 0.50; CI 95% 0.240–1.043; p 0.065). The estimated cumulative incidence curves for ANCA-negative and pANCA-positive subjects, adjusted for gender, age, comorbidities, and time of blood draw, are shown in **Figure 3**.

**Table 2:** Distribution of diagnosed cancer types among the study population.

Tumor location	Frequency	Percent
Leukemia / Lymphoma / MDS	7	31.8
Breast	6	27.27
Brain	4	18.18
Lung	4	18.18
Other tumors	2	9.09
Lung	2	9.09
Prostate	2	9.09
Retroperitoneum / Peritoneum	2	9.09
Pancreas	1	4.55
Head and neck	1	4.55
Stomach	1	4.55
Pleura	1	4.55
Skin	1	4.55
Melanoma	1	4.55
Liver	1	4.55
Pharynx	1	4.55
Thyroid	1	4.55
Total	38	100

The table provides an overview of tumor locations and their relative frequencies. MDS: Myelodysplastic Syndromes.

When patients with AAV (n=28) were excluded from the analysis, competing-risks regression continued to show no significant alteration in tumor risk (HR 0.53; 95% CI 0.24–1.16).

## Discussion

In this study, we investigated a cohort of subjects who were tested by various specialists over a period of 10 years for serum ANCA, likely due to suspected AAV. From this cohort, we selected 214 ANCA-positive subjects and 319 matched ANCA-negative controls, applying a propensity score-based matching algorithm that included comorbidities alongside age, gender, and time of blood draw.

Our primary aim was to determine the incidence of cancer while accounting for competing risks. Competing-risks regression, adjusted for gender, age, time of blood draw, and balanced comorbidity categories, showed no relationship between the presence of pANCA and the development of cancer, particularly hematological malignancies. These findings support the current belief that the increased cancer risk observed in AAV is primarily related to exposure to cyclophosphamide and other immunosuppressive therapies.

Recent findings suggested a role of systemic inflammation in AAV overall burden. Moon et al. showed that the C-reactive protein to serum albumin ratio - a composite index of systemic inflammation - is an independent predictor of all-cause mortality in patients with AAV (19). This association has been explained with two complementary hypotheses: first, an increased inflammatory burden at diagnosis contributing to increased immunosuppressive drug requirements or resistance and thereby an increased risk of treatment-related complications such as infections and malignancies. Secondly, patients with higher inflammation markers may have organ damage at baseline or develop complications during the follow-up, such as end-stage renal disease or diffuse alveolar hemorrhage, ultimately increasing mortality (19).

Moreover, chronic systemic inflammation has long been implicated in carcinogenesis by promoting oxidative stress, angiogenesis, and immune dysregulation (20). In line with this, elevated CRP levels have been associated with increased cancer risk and poor prognosis in several malignancies (20, 21).

However, despite these associations, current evidence does not support a direct oncogenic role for AAV-related vascular inflammation. A comprehensive review of the literature has focused on other mechanisms that might increase cancer risk in AAV patients and found contradictory evidence regarding a possible shared pathogenesis, concluding that suspicion of a neoplastic process should not be increased in AAV patients (15). Another study found similar results (16). In such a complex scenario, the theoretical ambivalent role of PR3 in GPA and acute myeloid leukemia is noteworthy. In a previous publication, we evaluated overall survival in patients without AAV by stratifying them with cANCA, pANCA or the absence of ANCA. No association was found between mortality and anti-MPO or anti-PR3 after adjusting for confounders, such as age, gender, and time of blood draw (17). ​​The present study builds upon that work by specifically investigating incident malignancy as a separate clinical outcome.

### Strength and Limitations

This study has a number of noteworthy advantages. In the first place, it is one of the largest cohorts to date investigating the association between cancer risk and ANCA positive in individuals who do not have ANCA-associated vasculitis. Second, the mean follow-up period nearly 4 years, allowing for the observation of medium-term outcomes. Third, by controlling for important confounders including comorbidities, a 1:2 matching method based on propensity scores helps guarantee balanced comparison groups. These methodological features enhance the internal validity and generalizability of our findings within the study setting.

Several limitations of our study should also be acknowledged. The complete information on vital status and cancer diagnoses was only accessible through the regional health information systems—namely the Lombardia Regional Health Registry and the ICH Cancer Diagnosis Registry. These sources do not routinely capture data from other Italian regions. Therefore, including out-of-region patients would have led to significant missing data for the primary and secondary endpoints (cancer incidence and survival), undermining internal validity. Moreover, restricting the cohort to Lombardia ensured not only consistency in data availability but also helped define a geographically and administratively coherent population. Lombardia is Italy’s most populous and medically advanced region, with one of the highest hospital care volumes in the country. While this may limit generalizability to other settings, we believe it enhances internal consistency and follow-up completeness. Importantly, by restricting the cohort to the Lombardia region, we also sought to reduce potential detection bias and surveillance bias. Second, we did not include data about previous exposure to smoking or drugs. Similarly, data about occupational exposures associated with ANCA, such as silica, were also unavailable. Nevertheless, it is reasonable to assume that these covariates are equally distributed between ANCA positive and negative in large groups. Furthermore, although our cohort is, to our knowledge, the largest investigated in relation to cancer outcomes in this context, the sample size remains limited in absolute terms. Therefore, it is necessary to conduct further studies to investigate the effect of ANCA on cancer mortality, particularly in larger populations of immunological patients. It would be particularly interesting to evaluate whether our results could be replicated in a prospective cohort study, with careful tracking of all possible confounders and mediators of effect. This study was specifically formulated to investigate an increased risk of cancer following pre-existing ANCA positivity. For this reason, patients with a known history of cancer were excluded. As such, the study does not investigate the opposite situation (development of ANCA in people with existing cancers)—a clinically important issue that may need to be considered also in the future. Another limitation concerns the statistical power of our matched cohort analysis. Based on the actual sample size and event rate after matching, we recalculated that the study had 80% power (α = 0.05) to detect a sub-hazard ratio of 1.27 or higher. This threshold represents a relatively small effect size, and values below this may have gone undetected. Notably, the 95% confidence intervals around the observed SHRs were wide (e.g., 0.24–1.04 for pANCA), encompassing both potential protective and harmful effects. Therefore, while our results do not support a statistically significant association between ANCA positivity and cancer risk, the study may have been underpowered to detect modest but clinically meaningful associations. Future research with larger sample sizes or pooled datasets might be required to more accurately estimate the true effect size, so these results should be interpreted cautiously. The exclusion of cANCA-positive patients from the competing-risk regression model was based on the absence of observed cancer cases in this group. However, this subgroup included only 44 individuals, and the absence of events should not be interpreted as evidence of no risk. We acknowledge that the limited size of the cANCA group precludes robust conclusions, and future studies with larger cANCA-positive cohorts will be needed to explore this potential association more definitively.

In conclusion, our findings suggest that isolated ANCA presence is not associated with an increased incidence of malignancies.

## Data Availability

The raw data supporting the conclusions of this article will be made available by the authors, without undue reservation.
