# Supplementary material for: Correlation between serum ANCA and cancer: a decade-long retrospective analysis of an Italian cohort
Source: Front Immunol. 2025 Aug 27;16:1631498. doi: 10.3389/fimmu.2025.1631498 (PMC12420293; doi:10.3389/fimmu.2025.1631498)
Supplement: Supplementary file 1 [file Table1.docx]

**Supplementary Material**

**Supplementary Table S1. Categorization of Comorbidities by Organ System.**

This table presents the distribution of patients with tumors stratified by ANCA status. For each group (ANCA-negative, cANCA-positive, and pANCA-positive), the table reports the number of tumor cases, the total number of patients at baseline, and the total person-years at risk. These data provide context for the incidence and time-to-event analyses across different ANCA profiles. Notably, no tumors were observed in the cANCA-positive subgroup.

| **Category** | **Included Conditions** |
| --- | --- |
| **1. Cardiovascular Diseases** | Myocardial infarction, STEMI, NSTEMI, Coronary syndrome, Ischemic heart disease, Cardiac arrest, Cardiomyopathy, Heart failure (reduced, preserved, intermediate EF), Congestive heart failure, Pulmonary edema, Pleural effusion, Coronary artery occlusion, Cardiogenic shock |
| **2. Respiratory Diseases** | COPD, Chronic bronchitis, Bronchiectasis, Asthma (including allergic), Emphysema, Pulmonary fibrosis, Cystic fibrosis, Pneumoconiosis (including asbestosis, silicosis, coal dust), Sarcoidosis, ARDS, ILD, Pulmonary embolism, Pulmonary hypertension, Farmer’s lung, Pneumonia, Tuberculosis |
| **3. Metabolic and Endocrine Disorders** | Type 1 and Type 2 diabetes mellitus, Diabetic nephropathy, neuropathy, retinopathy, foot ulcers, metabolic syndrome, Diabetic coma (including hyperosmolar, ketoacidosis), Obesity |
| **4. Kidney and Liver Diseases** | Chronic kidney disease, Nephrotic/nephritic syndrome, Glomerulonephritis, Polycystic kidney disease, Dialysis, Hepatic steatosis, Cirrhosis (including biliary), Hepatitis (viral, autoimmune), PSC, PBC, NASH, Ascites, Hepatorenal syndrome, Portal hypertension, Hepatic encephalopathy, Liver abscess, Liver granulomatosis, Wilson’s disease, Hemochromatosis |
| **5. Neurological Disorders** | Stroke, TIA, Cerebral ischemia/infarction, Intracerebral/subdural hemorrhage, Cognitive impairment, Dementia (including Alzheimer’s, vascular, frontotemporal, Lewy body), Parkinson’s disease, Multiple system atrophy, Epilepsy, Encephalopathy, Moyamoya disease, Aneurysm, Motor deficits, Delirium |
| **6. Autoimmune and Inflammatory Diseases** | Rheumatoid arthritis, Psoriatic arthritis, Systemic lupus erythematosus (SLE), Sjögren’s syndrome, Systemic sclerosis, Vasculitis (including GPA, MPA, Takayasu), Inflammatory bowel disease (Crohn’s disease, ulcerative colitis), Autoimmune hepatitis, Dermatomyositis, Polymyositis, Antiphospholipid syndrome, Sarcoidosis, Hashimoto’s thyroiditis, Graves’ disease, Myasthenia gravis, Autoimmune encephalitis, Uveitis, Alopecia areata, Pyoderma gangrenosum |

**Supplementary Table S2. Covariate Balance Before Matching – pANCA vs ANCA-negative**

*Results of standardized mean differences and t-tests comparing pANCA-positive and ANCA-negative patients before propensity score matching.*

| **Variable** | **Mean (Treated)** | **Mean (Control)** | **% Bias** | **t** | **p-value** | **Variance Ratio (T/C)** |
| --- | --- | --- | --- | --- | --- | --- |
| Age at blood draw (etax) | 65.30 | 63.14 | 12.6 | 1.30 | 0.194 | 1.05 |
| Male sex (gender_bin) | 0.379 | 0.357 | 4.3 | 0.45 | 0.653 | — |
| Time since blood draw (days) | 2663.1 | 2749.3 | –7.5 | –0.80 | 0.426 | 1.17 |
| Cardiovascular diseases | 0.126 | 0.114 | 3.4 | 0.37 | 0.711 | — |
| Respiratory diseases | 0.280 | 0.231 | 10.9 | 1.16 | 0.246 | — |
| Metabolic comorbidities | 0.210 | 0.196 | 3.4 | 0.36 | 0.719 | — |
| Kidney and liver diseases | 0.238 | 0.201 | 8.8 | 0.93 | 0.351 | — |
| Neurological diseases | 0.145 | 0.119 | 7.2 | 0.78 | 0.433 | — |
| Autoimmune/inflammatory diseases | 0.332 | 0.329 | 0.5 | 0.05 | 0.959 | — |

**Overall Matching Quality Statistics**

| **Pseudo R²** | **LR χ²** | **p > χ²** | **Mean Bias (%)** | **Median Bias (%)** | **B (%)** | **R** | **% of Variables with Var Ratio Outside [0.76–1.31]** |
| --- | --- | --- | --- | --- | --- | --- | --- |
| 0.007 | 4.19 | 0.899 | 6.5 | 7.2 | 19.8 | 1.07 | 0% |

- *% Bias: Standardized difference in means between treated and control group. Values >10% may indicate imbalance.*
- *Variance Ratio (T/C): Ratio of variances in treated vs control group. Ideally between 0.76 and 1.31.*
- *B: Maximum standardized bias; R: Ratio of variances of the propensity score in treated vs. control group.*
- *Pseudo R² and LR χ²: Overall covariate imbalance before matching. Smaller values indicate better balance.*
